# Fungal alkaline proteases and their potential applications in different industries

**DOI:** 10.3389/fmicb.2023.1138401

**Published:** 2023-03-30

**Authors:** Kadambari Subhash Pawar, Paras Nath Singh, Sanjay Kumar Singh

**Affiliations:** ^1^National Fungal Culture Collection of India (NFCCI), Biodiversity and Palaeobiology Group (Fungi), MACS’ Agharkar Research Institute, Pune, Maharashtra, India; ^2^Savitribai Phule Pune University, Pune, India

**Keywords:** alkaline proteases, classification, fermentation, alkaliphilic fungi, pH

## Abstract

The consumption of various enzymes in industrial applications around the world has increased immensely. Nowadays, industries are more focused on incorporating microbial enzymes in multiple processes to avoid the hazardous effects of chemicals. Among these commercially exploited enzymes, proteases are the most abundantly used enzymes in different industries. Numerous bacterial alkaline proteases have been studied widely and are commercially available; however, fungi exhibit a broader variety of proteases than bacteria. Additionally, since fungi are often recognized as generally regarded as safe (GRAS), using them as enzyme producers is safer than using bacteria. Fungal alkaline proteases are appealing models for industrial use because of their distinct spectrum of action and enormous diversity in terms of being active under alkaline range of pH. Unlike bacteria, fungi are less studied for alkaline protease production. Moreover, group of fungi growing at alkaline pH has remained unexplored for their capability for the production of commercially valuable products that are stable at alkaline pH. The current review focuses on the detailed classification of proteases, the production of alkaline proteases from different fungi by fermentation (submerged and solid–state), and their potential applications in detergent, leather, food, pharmaceutical industries along with their important role in silk degumming, waste management and silver recovery processes. Furthermore, the promising role of alkali–tolerant and alkaliphilic fungi in enzyme production has been discussed briefly. This will highlight the need for more research on fungi growing at alkaline pH and their biotechnological potential.

## 1. Introduction

**Figure 1:**
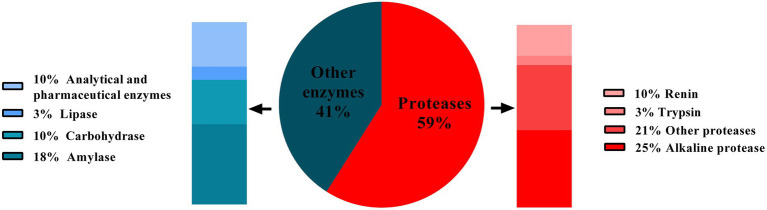
Contribution of types of proteases (red portion) in annual sales of the total enzyme. Source: adapted and modified from reference (Rao et al., 1998).

Enzymes are biocatalysts and are involved in nearly all biological reaction. Enzymes have been used in beer, wine, vinegar production, and cheese making since prehistoric time. The enzymes used in these processes were not pure and well–characterized. They were generally produced by micro–organisms that were spontaneously growing. Later, selected strains of micro–organisms were being used to produce enzymes on a large scale, followed by their purification. This development has made a remarkable contribution to the rectification of industrial processes. Further development of industrial enzymes has been revolutionized through directed mutation, protein engineering, and genetic engineering (Sharma, 2019). Currently, various enzymes are used in industries like lipases, proteases, amylases, cellulases, xylanases, etc. However, proteases remain the dominant type of enzyme as they are extensively valuable for multiple processes in detergents, dairy, food, paper, and pulp industries. Proteases hold about 60% shares of total enzymes sold commercially every year (Rao et al., 1998; Savitha et al., 2011; Figure 1). In 2019, the worldwide protease market was 2.76 billion USD, and expected to increase over the period of 2019–2024 with the annual growth rate of 6.1% (Choudhary et al., 2022). Among various proteases, alkaline proteases contribute the largest sector of the enzyme market, especially in the detergent industry (Abidi et al., 2011). Alkaline proteases are active at neutral to alkaline pH range and require either Asp-His-Ser triad (serine protease) or metal ions (metalloprotease) to act on the substrate (Choudhary et al., 2022). Alkaline proteases are preferred over the other types of proteases because of their ability to sustain under the alkaline pH without losing the action specificity. Owing to this fact, they have long been used in various industries, exclusively in the detergent industry. As an excellent and diverse enzyme reservoir, micro–organisms are extensively used for enzyme production to meet the current demand for proteases in various industries. Consequently, researchers keep searching for novel micro-organisms secreting alkaline proteases with desirable properties (Matkawala et al., 2021). In 1971, the first report was published regarding the production of alkaline protease by bacterium (*Bacillus* sp. strain 221) (Horikoshi, 1999). Since then, several bacterial and fungal genera have been studied for alkaline protease activity and exploited for their commercial production. In industrial processes, fungal proteases are the choice of enzymes over bacterial enzymes as they can be produced using low–cost substrate coupled with high and rapid productivity (Naeem et al., 2022). One more advantage of fungi over bacteria is biomass separation from production media; the mycelia can be easily removed from the broth, simplifying downstream processes (Souza et al., 2015; Arya et al., 2021). Therefore, the demand for fungal proteases for their extensive applications in various industries is increasing worldwide. The present review article deals with fungal alkaline proteases, their production, characterization, and applications in different fields.

## 2. Classification of proteases

In accordance with the Nomenclature Committee of the International Union of Biochemistry and Molecular Biology (IUBMB), proteases belong to subgroup 4 of hydrolases (group 3) (Barrett, 1997). The proteases have been primarily categorized based on three essential criteria: (1) site of action; (2) chemical nature of the active site; (3) evolutionary relationship with respect to their structure. On the basis of site of action, proteases are classified as exo–peptidases and endo–peptidases. Proteases acting near the termini of the polypeptide chain are placed under the group of exo–peptidases. In contrast, endo–peptidases act on the peptide bond in the internal region of the polypeptide chain, as free amino acid or carboxyl group negatively affects the enzyme activity. Exo–peptidases are further clustered into carboxypeptidases (acting near C terminus) and aminopeptidases (acting near N terminus). Based on the chemical nature of the active site, proteases were previously grouped into four major groups: serine proteases, cysteine proteases, aspartic proteases and, metalloproteases (Rawlings and Barrett, 1993). After discovering three more types of proteolytic enzymes, (1) threonine protease, (2) glutamic protease and, (3) asparagine peptide lyase, the hierarchy of proteolytic enzyme was made in 1993 was redefined. As per the online database for peptidases and their inhibitors (MEROPS), seven types of proteolytic enzymes have been described (Rawlings et al., 2018). Serine proteases contain serine residue at their catalytic site used for their catalytic activity (Polgár, 2005). They show their activity at both neutral and alkaline pH with an optimum range of 7.0 to 11.0. Serine proteases that are effective at highly alkaline pH are clustered as serine alkaline proteases, which is a major subclass of serine proteases with optimum pH around 10. Subtilisin of bacterial origin is the second–largest subclass of serine proteases effective at alkaline pH (Rao et al., 1998; Ekici et al., 2008). Second group, Cysteine proteases, also known as thiol proteases contain catalytic triad of amino acids, Cys-His-Asn (Verma et al., 2016). They are optimally active at neutral pH (Barrett and Rawlings, 2001). The third group, Aspartic proteases, has a dyad of two highly conserved aspartic acid residues (Friedman and Caflisch, 2010). Being maximally active at low pH (3.0–4.0), they are usually known as acidic proteases. Fourth group, Metallo–proteases are known for a characteristic of their obligation for divalent metal ion (zinc, cobalt, copper, nickel, manganese, and iron) to activate water which acts as a nucleophile to catalyze the reaction (Rawlings and Barrett, 1995). In 1995, threonine protease was discovered as the fifth catalytic type of protease while resolving the proteasome structure from *Thermoplasma acidophilum*. Out of fourteen, three subunits of peptidase of proteasome had N–terminal threonine that participated in catalysis by acting as a nucleophile (Brannigan et al., 1995; Seemüller et al., 1995). The sixth type of protease was identified in 2004 as glutamic peptidases. The catalytic site of glutamic protease has a catalytic dyad of glutamic acid and glutamine (Fujinaga et al., 2004). In 2011, a new type of proteolytic enzyme termed asparagine peptide lyase was discovered, which uses asparagine residue of their active site for peptide bond cleavage. The active site of this enzyme contains Asp-Asn amino acids. Unlike all other proteolytic enzymes, this type of enzyme does not require hydrolysis (use of water molecule for lysis) to catalyze the reaction. Hence, they are not peptidase but peptide lyase (Rawlings et al., 2011). Detailed classification of these proteolytic enzymes according to the MEROPS database has been summarized in Table 1 (Fernandes et al., 2023).

**Table 1:** Classification of proteolytic enzymes.

Type of proteolytic enzymes	Amino acids involved in active site	Mode of action*	Examples
(A) Exopeptidase			
(1) Aminopeptidase	–		–
Dipeptidyl peptidase (3.4.14)	–	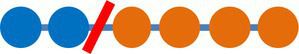	–
Tripeptidyl peptidase (3.4.16–3.4.18)		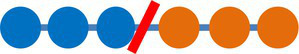	
(2) Carboxypeptidase		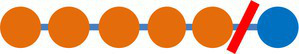	–
Dipeptidase (3.4.19)	–	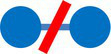	–
Peptidyl dipeptidase	–	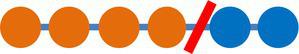	–
Serine type carboxypeptidase	Ser-Asp-His		–
Metallocarboxypeptidase	–		–
Cysteine type carboxypeptidase (3.4.15)	–		–
(B) Endopeptidase		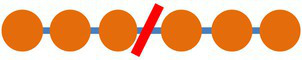	
Serine endopeptidase (3.4.21)	Asp-His-Ser		Chymotrypsin, trypsin, subtilisin
Cysteine endopeptidase (3.4.22)	Cys-His-Asn		Papain
Aspartic endopeptidase (3.4.23)	Asp-Asp		Pepsin, chymosin
Metalloendopeptidase (3.4.24)	His-His-Glu		Gelatinase, collagenase
Threonine endopeptidase (3.4.25)	N–terminal Thr		Proteasome endopeptidase complex
Endopeptidases of unknown catalytic mechanism (3.4.99)	–		–

The meaning of symbol * is as follows : Blue balls represent terminal amino acids; Brown balls represent amino acid residues from the internal polypeptide region. Slanting Red line indicated the site of cleavage.

Based on the evolutionary relationship, proteases are divided into different clans (also known as super families) that represent various protease families sharing a common ancestor in the evolution process (Argos, 1987). As per the recent data available on MEROPS database (Rawlings et al., 2018), proteolytic enzymes have been divided into 271 families, which are assembled into 56 clans. As of now, 53 families of serine peptidases, 96 families of cysteine peptidases, 16 families of aspartic peptidases, 76 families of metallopeptidases, 6 families of threonine peptidases, 2 families of glutamic peptidases, 10 families of asparagine lyases, 2 families of mixed catalytic type and 10 of unknown catalytic type have been identified (Rawlings and Bateman, 2019).

## 3. Production of fungal alkaline proteases

Alkaline proteases can be synthesized on a bulk scale using fungal isolates by submerged (Smf) and solid–state fermentation (SSF). Different fungi have been reported showing alkaline protease production potential (Table 2). Various cost–effective substrates like wheat bran, oil seed cakes, soybean bran, rice bran have been reported to give a considerable yield of the enzyme under SSF (Germano et al., 2003; Agrawal et al., 2004; Xiao et al., 2015; Novelli et al., 2016; Ram and Yepuru, 2018; Rukmi and Purwantisari, 2020). Although SSF offers volumetric productivity using cheaper substrates, superior monitoring and process control are still associated with Smf. Among fungi, *Aspergillus* sp. was found to be more prominent in the production of alkaline proteases. In a study, the regulation of alkaline protease production has been analyzed using both wild–type and mutant *Aspergillus nidulans*. This study describes an experiment to show the effect of carbon, nitrogen, and sulfur compounds on enzyme production regulation; when any of these compounds in the medium is withheld (Cohen, 1973). In another report, alkaline protease obtained from the cultures of *Aspergillus niger* strain Z1 in Czapek dox medium containing 1% casein can withstand the temperature up to 90°C and retained 48.4% of its original activity for 15 min. This alkaline protease has been reported to have its optimum activity at temperature 40°C and pH 9.0 (Coral et al., 2003). *Aspergillus tamarii* was tested for alkaline protease production, and its growth conditions were optimized in solid–state fermentation using wheat bran and submerged fermentation to maximize yield. The isolate was found to produce maximum enzyme at pH 9.0 and temperature 30°C in both fermentation types and required 65% moisture content in wheat bran (Anandan et al., 2007). Moreover, in the first report aimed to optimize production of alkaline protease by *Aspergillus clavatus*, it has been reported that the enzyme was highly active at pH 9.5 and 40°C temperature. The isolate showed the highest proteolytic activity (38 U/mL), when the medium was supplemented with 1% glucose and casein. Among tested nitrogen sources, NH_4_NO_3_ (0.2% w/v) was reported as the best nitrogen source and showed 27 U/mL enzyme activity (Tremacoldi and Carmona, 2005).

**Table 2:** List of fungi producing alkaline proteases and characteristics of proteases.

Genus	Fungal isolate	Fermentation type	Optimum pH	Optimum temperature	Substrate	Purification step	Type of protease	References
*Aspergillus*	*Aspergillus niger* Z1	Smf	9.0	40°C	Czapek Dox medium	Ethanol precipitation	Serine protease	Coral et al. (2003)
	*Aspergillus clavatus* ES1	Smf	8.5	50°C	CaCl_2_.7H_2_O, KH_2_PO_4_, Na_2_HPO_4_, MgSO_4_.7H_2_O, ZnCl_2_, NaCl, whole *Sardinella* (*Sardinella aurita*) fish flour, wheat bran	Acetone treatment, Sephadex G-100 gel filtration, CM-Sepharose separation	Serine protease	Hajji et al. (2007)
	*Aspergillus tamarii* NRRL 20818	SSF	9.0	30°C	Wheat bran	Ammonium sulphate precipitation, DEAE-cellulose, DEAE-cellulose	Serine protease	Anandan et al. (2007)
		Smf	7.0–10.0	30°C	Glucose, peptone, skimmed milk, yeast extract, Na_2_HPO_4_, NaNO_3_			
	*Aspergillus parasiticus*	SSF	8.0	40°C	Wheat bran	Acetone precipitation, DEAE-Sephadex A-50 Flow through, Gel filtration (FPLC)	Serine protease	Tunga et al. (2003)
	*Aspergillus terreus* (IJIRA 6.2)	SSF	8.5	37°C	Wheat bran	DEAE-Sephadex A25, phosphocellulose column, hydroxyapatite column, casein-Sepharose column, Sephacryl-S-300 column	Serine protease	Chakrabarti et al. (2000)
	*Aspergillus ochraceus* BT21	Smf	9.0	50°C	Dextrin, peptone, K_2_HPO_4_, MgSO_4_, KCl, FeSO_4_	Sephacryl S-200 gel filtration chromatography, Ion-exchange chromatography	Serine protease	El-Khonezy et al. (2021)
*Trametes*	*Trametes cingulata* CTM10101	Smf	9.0	60°C	Potato dextrose broth	Ammonium sulphate precipitation, Fast protein liquid chromatography	Serine protease	Omrane Benmrad et al. (2016)
*Trichoderma*	*Trichoderma atroviride F6*	Smf	8.0–9.0	50°C	whole-feather medium	Ammonium sulphate precipitation, DEAE–cellulose column	Serine protease	Cao et al. (2008)
	*Trichoderma longibrachiatum*	Smf	9.0	40°C	KH_2_PO_4_, Na_2_HPO_4_, CaCl_2_.7H_2_O, MgSO_4_.7H_2_O, NaCl, ZnCl_2_, different agro-industrial products	Ammonium sulphate precipitation	Unidentified	Chandra Behera et al. (2021)
*Penicillium*	*Penicillium* sp.	SSF	9.0	45°C	Wheat bran	–	Unidentified	Agrawal et al. (2004)
	*Penicillium chrysogenum* X5	Smf	10.0	80°C	Yellow lentil flour, tryptone, glucose, CaCl_2_, KH_2_PO_4_, K_2_HPO_4_, trace elements	Ammonium sulphate precipitation, UNO Q-12 FPLC	Serine protease	Benmrad et al. (2018)
	*Penicillium rubidurum*	Smf	8.0	40°C	KH_2_PO_4_, Na_2_HPO_4_, CaCl_2_.7H_2_O, MgSO_4_.7H_2_O, NaCl, ZnCl_2_, different agro-industrial products	Ammonium sulphate precipitation	Unidentified	Chandra Behera et al. (2021)
*Fusarium*	*Fusarium* sp. BLB	Smf	9.5	50°C	soybean powder, glucose, polypepton, yeast extract, KH_2_PO_4_, MgSO_4_	Ammonium sulphate precipitation, CM-Toyopearl 650 M column elution, Superdex 75 HR 10/30 column	Serine protease	Ueda et al. (2007)
*Ophiostoma*	*Ophiostoma piceae* 387 N	Smf	7.0–9.0	40°C	CaCl_2_.2H_2_O, KH_2_PO_4_, Na_2_HPO_4_, MgSO_4_.7H_2_O, potassium hydrogen phthalate	hydrophobic interaction chromatography, Ammonium sulphate precipitation	Unidentified	Abraham and Breuil (1996)
*Myceliophthora*	*Myceliophthora* sp.	SSF	9.0	50°C	Wheat bran	–	Unidentified	Zanphorlin et al. (2010)
		Smf	7.0	50°C	Casein, (NH_4_)_2_SO_4_, MgSO_4_.7H_2_O, NH_4_NO_3_			
*Engyodontium*	*Engyodontium album* BTMFS10	SSF	11.0	60°C	Wheat bran	Ammonium sulphate precipitation, Ion-exchange chromatography (DEAE)	Unidentified	Chellappan et al. (2006)
*Clonostachys*	*Clonostachys rosea*	Smf	9.0–10.0	60°C	Glucose, gelatin, peptone, yeast extract	Ammonium sulphate precipitation, HiPrep Phenyl FF column, SOURCE 15Q	Serine protease	Li et al. (2006)
*Botrytis*	*Botrytis cinerea*	Smf	8.0	50°C	Yeast extract, glucose, gelatin/soy protein	Ammonium sulphate precipitation, Superdex G-75 gel filtration, Anion-exchange chromatography with SP-Sepharose	Unidentified	Abidi et al. (2011)
*Beauveria*	*Beauveria* sp. MTCC 5184	Smf	9.0	50°C	Glucose, yeast extract, mustard seed cake	Ammonium sulphate precipitation, DEAE-cellulose column	Serine protease	Shankar et al. (2011)
*Conidiobolus*	*Conidiobolus coronatus* ATCC PTA–4132	Smf	9.0	28°C	MGYP broth	–	Unidentified	Laxman et al. (2005)
*Microsporum*	*Microsporum canis* strain IHEM 10157	Smf	9.0	55°C	Cat keratine, Glucose, inositol, pyridoxine, thiamine	Affinity based chromatography using bacitracin agarose	Subtilisin-like serine protease	Mignon et al. (1998)
*Chrysosporium*	*Chrysosporium keratinophilum*	Smf	9.0	90°C	keratin suspension, lactose, MgSO_4_.7H_2_0, FeSO_4_.7H_2_0, ZnSO_4_.7H_2_0, peptone	cold-acetone precipitation, gel-filtration on Sephadex G-75	Unidentified	Dozie et al. (1994)

Further a report is available based on activity of bleach stable alkaline protease by the newly isolated *Aspergillus clavatus* ES1 (Hajji et al., 2007). The enzyme showed a 7.5–fold rise in specific activity when purified by acetone precipitation, gel filtration (Sephadex G–100), and ion–exchange chromatography (CM–Sepharose), with 29% recovery having optimum activity at 50°C and pH 8.5. In another study, alkaline protease obtained from *Aspergillus terreus* was studied for its use in detergent formulation. This monomeric enzyme with molecular weight 16 ± 1 kDa showed 148.9 U/mg enzyme activity. Casein was the best substrate over gelatin with 12.8 U/mL Vmax and 5.4 mg/mL Km. The enzyme was active from pH 8.0–12.0, showing optimum activity at pH 11.0 (Niyonzima and More, 2015). Additionally a literature is available (Ke et al., 2018) in which the successful expression of truncated alkaline protease in Pichia pastoris KM71 from *Aspergillus sojae* GIM3.33 using the RT–PCR technique has been described. This recombinant alkaline protease had 400.4 ± 40.5 U/mL enzyme activity up to three days after induction using methanol and was optimally active at pH 10.0 and temperature 45°C. A recent study was focused on the production of alkaline protease using *Aspergillus* sp. isolated from an Ethiopian food, Injera, and the use of response surface methodology (RSM) for process optimization. RSM optimization found that pH 8.24, 30.5°C, and 0.316% sucrose concentration gave the maximum enzyme activity (166.4221 U/mL) (Mustefa Beyan et al., 2021).

Besides *Aspergillus*, very few other fungal genera have been reported for alkaline protease production. In a study, assessment of the protease production by *Fusarium oxysporum* f. sp. *dianthi* race 2 (Fod) using cell wall extracts of susceptible and resistant cultivars of carnation (*Dianthus caryophyllus* L.) has been described (Rodríguez et al., 2017). This study was aimed to reveal the protease–assisted pathogenicity in Fod–carnation interaction. In the presence of cell wall extract of susceptible carnation, Fod produced maximum protease enzymes. Serine protease (trypsin), one of the three isoenzymes secreted by the fungus, was further studied for its identification and biochemical characterization. The enzyme was highly active at pH 8.0 and 50°C temperature, which was then purified using gel filtration (Sephadex G–75), DEAE–cellulose ion exchange, and affinity chromatography (Benzamidine Sepharose 6B). The molecular weight of this protease was 54 kDa, showing Km of 0.31 mg/mL and Vmax of 24.7 μmol/min. Further an interesting study is available on alkaline protease produced by *Clonostachys rosea* (syn. *Gliocladium roseum*) in which the enzyme was studied for its role in infecting nematodes. Its biochemical characterization has been performed upon purification of enzyme using ammonium sulfate precipitation, HiLoad 16/10 Phenyl Sepharose FF column, and anion exchange column (SOURCE 15Q 4.6/100 PE column). The enzyme exhibited highest activity at 60°C temperature and pH 9.0–10.0. Nematicidal studies revealed that the crude enzyme was superior in nematode immobilization than the purified enzyme with 80 ± 5% nematicidal activity (Li et al., 2006). Further the fibrinolytic alkaline protease (M.W. 27 kD) produced from *Fusarium* sp. BLB isolated from plant leaf (*Hibiscus*) has been characterized (Ueda et al., 2007). Purification of the enzyme was carried out by (NH_4_)_2_SO_4_ precipitation and column chromatography (CM–Toyopearl 650 M and Superdex 75). The enzyme had its maximum fibrinolytic activity at pH 9.5 and temperature 50°C. In another study, from the culture of *Penicillium chrysogenum* X5 the thermostable serine alkaline protease was purified, and its biochemical characterization was done. The results revealed that the enzyme was active at 10.0 pH and 80°C temperature. Enzyme purification was done by following three steps which include heat treatment at 80°C/10 min, (NH_4_)_2_SO_4_ precipitation (30–50%) and dialysis, and anion exchange chromatography (UNO Q–12) using the FPLC system. To evaluate its compatibility with laundry detergents, the enzyme was tested for its wash performance by using it as an additive with detergents. Results revealed that, the enzyme improved the performance of laundry detergent to clean the egg and blood stains on the fabric (Benmrad et al., 2018). A new alkaline protease obtained from *Penicillium nalgiovense* showed its optimum activity at pH 8.0, 35°C temperature, and 0.25 M NaCl concentration. (NH_4_)_2_SO_4_ precipitation, dialysis, and ultrafiltration increased the enzyme activity by 12–fold. The molecular mass (45.2 kDa) was confirmed by ESI–MS analysis (Papagianni and Sergelidis, 2014). One more species of *Penicillium* has been studied for the production of alkaline protease using SSF (Xiao et al., 2015). From fermented fish sauce, 30 fungi were isolated based on morphology, among which only *P. citrinum* YL–1 showed enzyme activity. Under Plackett–Burman design, three significant variables like peptone concentration, initial pH, and moisture content were chosen for increasing enzyme yield. The influence of these variables on production of alkaline protease was studied using Box–Behnken design. Another study, focused on fungal isolate *Conidiobolus coronatus* from *Entomophthorales* order, showed the usage of alkaline protease for gelatin degradation and silver recovery from x–ray film. The obtained enzyme was active at 40°C and pH 9.0 with 1.35 U/mL specific activity (Shankar et al., 2010). *Scopulariopsis* spp. was used to produce alkaline protease with a molecular mass of 15 ± 1 kD showing 138.1 U/mg specific activity. The optimum pH of this enzyme was 9.0, while the enzyme was active and stable between the pH 8.0 to 12.0. The enzyme was optimally active at temperature 50°C. The enzyme had 4.3 mg/mL Km and 15.9 U/mL Vmax, using casein as a substrate. As the enzyme was found to be stable in the presence of surfactants, oxidizing and bleaching agents, active at high temperatures and alkaline pH, exhibiting wide range of substrate specificity and compatibility with detergents, its consumption in the detergent formulation has been highly recommended (Niyonzima and More, 2014).

## 4. Applications of alkaline proteases

Fungal alkaline proteases have a several applications, predominantly in the detergent and food industries. Fungal alkaline proteases are envisioned to have wide–ranging uses in other fields like bioremediation and leather treatment, etc. (Kumar and Takagi, 1999). Table 3 highlights some of the crucial uses of fungal alkaline proteases.

**Table 3:** Application of fungal alkaline proteases in different industries.

Fungal isolates	Application	References
*Conidiobolus brefeldianus*	Dehairing of skins/hides	Khandelwal et al. (2015)
*Conidiobolus coronatus* ATCC PTA–4132	Silver recovery from photographic film	Shankar et al. (2010)
*Penicillium* sp.	Soy protein hydrolysis	Agrawal et al. (2004)
*Aspergillus niger* LCF 9	Collagenolytic activity	Barthomeuf et al. (1992)
*Fusarium* sp. BLB	Fibrinolytic activity	Ueda et al. (2007)
*Aspergillus niger* DEF 1	Fibrinolytic activity	Lanka et al. (2017)
*Aspergillus strain* KH 17	Fibrinolytic activity	Palanivel et al. (2013)
*Scopulariopsis* spp.	Detergent formulation	Niyonzima and More (2014)
*Penicillium godlewskii* SBSS 25	Detergent formulation	Sindhu et al. (2009)
*Trametes cingulata* CTM10101	Detergent formulation	Omrane Benmrad et al. (2016)
*Graphium putredinis*, *Trichoderma harzianum*	Detergent formulation	Savitha et al. (2011)
*Aspergillus* sp. DHE7	Detergent formulation	Suresh and Dass (2022)
*Penicillium chrysogenum* X5	bio–additive for textile processing	Benmrad et al. (2018)
*Trichoderma longibrachiatum, Aspergillus niger*	Blood stain removal	Suryawanshi and Pandya (2017)
*Aspergillus oryzae* NRRL–447	Keratinolytic activity	Ali et al. (2011)
*Aspergillus* spp.	Keratinolytic activity	Kim (2003)
*Cunninghamella echinulata*	Keratinolytic activity	More et al. (2013)
*Fusarium oxysporum*	Keratinolytic activity	Prolo et al. (2020)
*Chrysosporium tropicum*	Keratinolytic activity	Koutb et al. (2022)
*Conidiobolus coronatus* (NCIM 1238)	to resolve the racemic mixtures of DL–phenylalanine and DL–phenylglycine	Sutar et al. (1992)

### 4.1. Detergent industry

Proteases are essential and standard additives in detergents, as they can remove all kinds of proteinaceous materials (Nehra et al., 2002). The first enzymatic formulation named “Brunus” was prepared in 1913, containing crude pancreatic extract and sodium carbonate (Razzaq et al., 2019). A detergent containing a bacterial enzyme with the trade name “BIO–40” was marketed for the first time in 1956 (Kumar et al., 2008). Proteases act against a wide range of substrates, making it worthwhile to remove stains of foodstuff, blood, and body secretions. Characteristics such as stability at alkaline pH and high temperature, ability to withstand with surfactants, oxidizing and chelating agents will make alkaline proteases an indispensable candidate for their application in the detergent industry (Sharma, 2019).

Many researchers studied the compatibility of alkaline protease with detergents to make it sound as a detergent additive. In a report it is presented that the alkaline protease produced by *Conidiobolus coronatus* (NCL 86.8.20) retained its 90% of activity at a lower concentration (0.05 mg/mL–1) in commercial detergent solution after 1 h of incubation at 40°C; suggesting its possible use in detergents (Phadatare et al., 1993). Alkaline serine–protease of *Aspergillus clavatus* ES1 showed extreme stability (100%) in non–ionic surfactant [5% (v/v) tween 80 and triton X–100]; however, enzyme retained its 90% of activity when tested with 0.1% SDS (strong anionic surfactant). Also, the enzyme showed moderate stability towards 1% (w/v) and 2% (w/v) sodium perborate, retaining 71 and 53% activity, respectively (Hajji et al., 2007). In another study, similar results were obtained for the protease produced by *Scopulariopsis* spp., where an increase in protease activity with Tween–80, SDS, and Triton X–100 with retention of 50% enzyme activity in the commercial detergents (enzymatic and nonenzymatic) was obtained (Niyonzima and More, 2014). Another alkaline protease of *Aspergillus* species (*A. niger* and *A. terreus*) retained 50–80% of its initial activity in the presence of various commercially available detergents such as Tide, Hattic, Savo, Surf, Henko, Persil, Wheel, and Aerial (Ali, 2008; Devi et al., 2008). Further a team of researchers studied the compatibility of enzymes produced by two fungal isolates, *Graphium putredinis, Trichoderma harzianum*, and an intergenetically developed fusant. All three enzymes were stable towards SDS and sodium perborate with retention of 58.25–73.82% and 61.58–70.24% of residual activity, respectively, while enzyme from fusant was highly stable than parents with 76.74% of activity when tested for its compatibility with commercial detergent Rin Advanced (Savitha et al., 2011). Similar studies were focused on the compatibility and incorporation of alkaline protease produced by *Aspergillus niger* and *Aspergillus* sp. DHE7 as an additive in detergent formulation (Pundir et al., 2012; El-Ghonemy and Ali, 2021). Recent literature highlighted the introduction of third-generation cold-adapted alkaline proteases to the detergent industry which were found to have excellent activity and stability in surfactants and bleaches (Suresh and Dass, 2022).

### 4.2. Leather industry

In leather–processing industries, alkaline proteases have extensive applications in various processes like soaking, debating, and depilating of skin and hides. This enzymatic treatment removes unwanted pigments, increases the skin area, and produces a clean hide (Arora, 2003). Alkaline proteases accelerate the dehairing process, as the attack of protease on the hair follicle at higher pH makes the hair removal process easy. Conventionally, this has been carried out using chemicals like a saturated solution of lime and sodium sulfide to treat an animal hide; but this is a very expensive and produces intensely polluting effluent (Anwar and Saleemuddin, 1998). A very few studies have been reporting the application of fungal alkaline protease in the leather industry. A study was aimed on the potential use of alkaline protease produced by *Aspergillus flavus* in the tannery as a depilation agent (Malathi and Chakraborty, 1991). Also, from experimental data, it was confirmed that enzymatic treatment increased the tensile strength, stitch tear strength, bursting strength of leather as compared to control. A similar study has reported using alkaline protease as a dehairing agent in tannery (Pal et al., 1996). Further, the potential use of protease obtained from *Conidiobolus coronatus* has been studied in soaking, dehairing, and bating animal skin/ hide (Khandelwal, 2013).

### 4.3. Food industry

The production of cheese is the primary application of proteases in the dairy industry. For good flavor and texture development, proteolysis plays a vital role (Fox, 1982). Protease hydrolyses kappa casein and stabilizes micelle formation, which prevents coagulation. Proteases obtained from fungi such as *A. oryzae, Rhizomucor miechie, R. pusillus* are extensively used as coagulants for cheese production in the dairy industry (Neelakantan et al., 1999). Besides the dairy industry, alkaline protease was used in meat tenderization because of its potential to hydrolyze connective tissue and muscle fiber (Ellaiah et al., 2002).

### 4.4. Pharmaceutical industry

The vast diversity and specificity of fungal proteases are significant advantages in developing effective therapeutic agents. Protease obtained from *Aspergillus oryzae* has been applied to cure the digestive disorders like lytic enzyme deficiency syndromes by its oral administration (Rani et al., 2012). Alkaline protease produced by *Aspergillus niger* LCF 9 is used for therapeutic application because of its collagenolytic activity (Barthomeuf et al., 1992). Fibrinogen, fibrin, and blood clot were hydrolyzed efficiently by proteases obtained from *Aspergillus* strains (El-Shora and Metwally, 2008). Treatments for illnesses including Dupuytren’s disease, Peyronie’s disease, wound healing, burns, glaucoma, intervertebral disc herniation, debridement, keloid, vitrectomy, and cellulite involve alkaline protease with collagenase activity (Gurumallesh et al., 2019). In burns and wound treatment, immobilized subtilisins have been formulated as soft gel-based formulae, ointments, and bandage materials (Matkawala et al., 2021).

### 4.5. Waste management

Keratin is the primary protein found in waste from the poultry and leather industry. Because of its compactly packed polypeptide, which is stabilized by strong disulfide bonds and some weak interaction, makes it difficult to degrade. Fungal alkaline proteases have been accessed by many researchers for the degradation of keratin. A study has been done on the degradation of keratin using proteases from five species of *Aspergillus* (*A. flavus, A. niger, A. fumigatus, A. terreus*, and *A. nidulans*). Among them, protease from *A. niger* degraded maximum keratin of chicken feathers with 28 μg/mL of cysteine release followed by *A. flavus, A. fumigatus, A. nidulans,* and *A. terreus* (Kim, 2003). Protease from *A. oryzae* has also been reported to degrade feather keratin with the release of various essential (threonine, isoleucine, methionine, leucine, tyrosine, valine, histidine, and lysine) and non–essential (glycine, serine, alanine, phenylalanine, glutamic acid, aspartic acid, and arginine) amino acids and ammonia. Released amino acids and ammonia from the waste can be further used as fertilizer or additives in feed (Ali et al., 2011). In another study, ten dermatophytes from the soil was isolated and studied to biodegrade human and animal hair. Among ten dermatophytes, *Chrysosporium indicum* degraded human hair with maximum weight loss (56.66%); whereas, *Microsporum gypseum* and *Trichophyton verrucosum* degraded animal hair with 49.34% weight loss (Sharma et al., 2011).

### 4.6. Silk degumming

The proteases are significant candidates in the silk industry for silk degumming or sericin removal from silk. Rough texture of the raw silk fiber is due to the presence of sericin in the peripheral region of fiber. Degumming of silk before dyeing, helps to improve the sheen, texture, and color of cloth. Various fungal proteases have been used for silk degumming. The literature is available on the comparative study of silk degumming using Marseille’s soap and enzyme from *Conidiobolus* species (Gulrajani et al., 2000). Fungal protease showed 19.8% weight loss after incubation of 3 h at 37°C, while degumming with Marseille’s soap gave 20.5% weight loss on the incubation of 1.5 h at boiling temperature. Successful degumming at a lower temperature will make enzymatic degumming a cost–effective process compared to chemical degumming. One more study based on a similar approach reported comparing degumming by six microbial proteases (4 fungal and 2 actinomycetes) and conventional method using alkali and soap. Among tested proteases of microbial origin, *Conidiobolus brefeldianus* MTCC 5185 and *Actinomycete*–2 (BOA–3) showed best results with 21.10 ± 0.67 and 21.78 ± 0.99% weight loss, respectively, in a short time which is similar to the weight loss (21.40 ± 0.75%) by conventional method (More et al., 2013).

### 4.7. Silver recovery

The photography industry uses a large quantity of silver in the preparation of light–sensitive emulsion. Used X–ray film has been found to contain around 1.5 to 2.0% silver in the gelatin layers. Silver recovery from X–ray films by conventional methods, mainly by combustion of X–ray films, causes pollution in environment. Hence, the hydrolysis of the gelatin present on the X–ray films using fungal alkaline proteases can be used as an alternative option for silver recovery (Sharma et al., 2019). In one of the studies, protease obtained from *Conidiobolus coronatus* showed 5% weight loss in x–ray film with the silver recovery of 3.87% (w/w) of sludge weight and 0.2% (w/w) of x–ray film weight, respectively (Shankar et al., 2010). Similarly from one more study, the recovery of 0.135 gm of silver from 40 gm of x–ray film has been reported with 0.335% yield using protease produced by *Aspergillus versicolor* PF/F/107, suggesting an eco–friendly way to the silver from used x–ray films (Choudhary, 2013).

## 5. Alkaliphilic/alkali-tolerant fungi as a potential source of alkaline enzymes

Primarily most of the fungal species are known to grow at weakly acidic to neutral pH and only handful of researchers has reported on the fungi which can grow at alkaline pH as well. Consequently, these groups of alkaliphilic and alkali–tolerant fungi have remained unexplored for their capability for production of commercially valuable products that are stable at alkaline pH.

A research done in Japan on the distribution of alkalophilic and alkali–tolerant fungi from the limestone caves soil in Japan revealed that approximately one–third (30.8%) of the isolates had the optimum pH in the alkaline range. Fungal species belonging to genera *Acremonium* and *Chrysosporium* were found to be predominant at alkaline pH. (Nagai et al., 1998). A recent study for understanding the evolution alkaliphily in fungi has described the diversity of weak alkali–tolerant to alkaliphilic fungi from soils around the basin of soda lakes in Asia and Africa. It shows the wide of alkaliphilic trait throughout ascomycota division of fungi. Fungi belonging to *Emericellopsis* lineage (*Emericellopsis alkalina*) of order *Hypocreales*, along with fungi from *Plectosphaerellaceae* (*Sodiomyces species, Acrostalagmus luteoalbus*), *Pleosporaceae* (*Alternaria* sect. *Soda*), *Chaetomiaceae* (*Thielavia* sp.) families, were found to be strong alkalitolerants and effective alkaliphiles. Moderate alkali–tolerant fungi included members of genus *Scopulariopsis*, *Fusarium*, *Cladosporium* and Acremonium–like species. While, *Penicillium* sp., *Purpureocillium lilacinum*, and *Alternaria alternata* species showed weak alkali tolerance (Grum Grzhimaylo et al., 2016). In another such study from Thailand, 490 fungi were isolated from various mycological samples, including tree–holes, roots, leaf litter, wood, and soil at pH 11. A total of 324 (66%) isolates were screened for alkaline enzymes like Arabinanase, amylase, potato–galactanase, and protease. This screening revealed that most of these alkali–tolerant fungal isolates were able to produce at least one of the listed enzymes, and a few strains were positive for the activity of all four enzymes (Kladwang et al., 2003).

This dig at the literature highlights the gap in research to produce alkaline enzymes, especially industrially important alkaline proteases from alkaliphilic and alkali–tolerant fungi. This will lead to the production of more promising alkaline enzymes and can ease the wide range of industrially important reactions that are done at alkaline conditions.

## 6. Conclusion

Global consumption of various enzymes, especially microbial proteases, in industrial applications is increasing because of their wide range of applications in different industries. To meet the existing demand and rapid and easy production, the exploitation of other alternative microbial sources like fungi becomes essential. Fungal proteases are emerging as the best alternative and possess numerous commercial applications. The present comprehensive review describes the alkaline proteases obtained from fungi of different genera and their potential applications in various industries. Meager studies of fungal alkaline proteases leave the scope for research in alkaline proteases from fungi for their industrial applications, which will be helpful for future research worldwide. This review also highlights the need to study the alkaliphily trait spread among fungi, the diversity of alkali-tolerant and alkaliphilic fungi, and their use for the production of various alkaline enzymes. Moreover, due to their intrinsic adaptation to withstand the alkalinity, these enzymes can be used as a promising alternative for current alkaline enzymes produced by fungi growing at acidic to neutral pH.

## Author contributions

All authors listed have made a substantial, direct, and intellectual contribution to the work and approved it for publication.

## Conflict of interest

The authors declare that the research was conducted in the absence of any commercial or financial relationships that could be construed as a potential conflict of interest.

## Publisher’s note

All claims expressed in this article are solely those of the authors and do not necessarily represent those of their affiliated organizations, or those of the publisher, the editors and the reviewers. Any product that may be evaluated in this article, or claim that may be made by its manufacturer, is not guaranteed or endorsed by the publisher.
